# Quantitative myocardial perfusion during stress using CMR is impaired in healthy Middle Eastern immigrants without CV risk factors

**DOI:** 10.1038/s41598-022-23131-x

**Published:** 2022-10-29

**Authors:** Robert Jablonowski, Louise Bennet, Henrik Engblom, Anthony H. Aletras, Hui Xue, Peter Kellman, Marcus Carlsson, Håkan Arheden

**Affiliations:** 1 Clinical Physiology, Department of Clinical Sciences Lund, Lund University Hospital, Lund University, Skane University Hospital, 221 85 Lund, Sweden; 2 Department of Clinical Sciences, Lund University, Malmö, Sweden; 3 Laboratory of Computing, Medical Informatics and Biomedical-Imaging Technologies, School of Medicine, Aristotle University of Thessaloniki, Thessaloniki, Greece; 4 National Heart-Lung and Blood Institute, Bethesda, MD USA

**Keywords:** Magnetic resonance imaging, Risk factors, Cardiovascular diseases

## Abstract

Middle Eastern immigrants constitute a growing proportion of the European population and compared to native Swedes are more insulin resistant, which can contribute to atherosclerosis. Quantitative first pass perfusion (qFPP) using cardiovascular magnetic resonance (CMR) can detect early signs of cardiovascular disease (CVD). The aim was to study if myocardial perfusion differs between healthy male Middle Eastern immigrants and native male Swedes. Eighteen Iraqi- and twelve Swedish born controls, all males, never smokers with no CVD risk factors were included. Global myocardial perfusion at rest and stress was assessed using qFPP and by phase-contrast CMR imaging of coronary sinus flow. Quantitative first pass perfusion analysis (mean ± SD) demonstrated no difference at rest between Iraqi and Swedish males (0.8 ± 0.2 vs 1.0 ± 0.4 ml/min/g, *P* = 0.38) but lower perfusion during adenosine in Iraqi males (2.9 ± 0.7 vs 3.5 ± 0.7 ml/min/g, *P* = 0.02). Myocardial perfusion assessed by coronary sinus flow demonstrated similar results with no difference in resting perfusion between groups (0.7 ± 0.2 vs 0.8 ± 0.2 ml/min/g, *P* = 0.21) but a lower perfusion during adenosine in the Iraqi group (3.0 ± 0.2 vs 3.7 ± 0.6 ml/min/g, *P* = 0.01. Myocardial perfusion during adenosine stress was lower in healthy Iraqi immigrants compared to Swedish controls suggesting impaired microvascular function and risk of underestimating CVD risk in healthy individuals of Middle Eastern origin.

## Introduction

Cardiovascular disease (CVD) is the most common cause of death in Europe and one of the strongest risk factors for CVD is type 2 diabetes^1^. Middle Eastern immigrants constitute a growing proportion of the European population and are at high risk for type 2 diabetes, obesity and hyperlipidemia^2^, translating to an increased risk for CVD^3^. In Sweden, one of the largest immigrant populations are from Iraq^4^. The Iraqi immigrant group in southern Sweden has been studied within the population based MEDIM (impact of Migration and Ethnicity on Diabetes in Malmö) study. The studies from MEDIM have previously demonstrated that Iraqi immigrants irrespective of diabetes related risk factors, are more insulin resistant compared to native Swedes, which can contribute to increased risk of atherosclerosis^5^. In a recent cohort study including new onset type 2 diabetes patients (the ANDIS study), it was shown that first generation Middle Eastern immigrants had a more insulin‐deficient diabetes phenotype and genotype than native Swedes, as well as a higher risk of macrovascular diabetic complications such as coronary events^6^. However, the pathophysiological basis for the increased risk of CVD in Middle Eastern immigrants is still not clear.

Coronary microvascular function, using positron emission tomography (PET), has been shown to be impaired in patients with the metabolic syndrome as well as in individuals at high risk for CVD without the metabolic syndrome^7^. Single-photon emission computed tomography (SPECT) can also be used for quantitative estimates of myocardial perfusion but there are inherent limitations as low tracer uptake at high flow rates^8,9^. Cardiovascular magnetic resonance (CMR) imaging conducted without radioactive tracers, offers superior morphological and functional characterization of the myocardium compared to SPECT and PET and the possibility to study both microvascular function by quantitative (ml/min/g) first pass perfusion (qFPP)^10^ and extracellular volume mapping^11,12^. Quantitative first pass perfusion has been integrated inline in the clinical work flow and validated against PET in patients^10,13^.

In the current study we hypothesized that healthy Middle Eastern immigrants from Iraq living in Sweden without traditional CVD risk factors have altered myocardial microvascular function compared to native Swedes. Since there are sex differences in cardiometabolic risk, and males are at higher CVD risk than females, the current study was performed on males only. Therefore, the aim of the current study was to investigate if quantitative myocardial perfusion using CMR differs between male Iraqi immigrants, without cardiometabolic disease and native male Swedish controls.

## Methods

### Study population and study design

**Figure 1:**
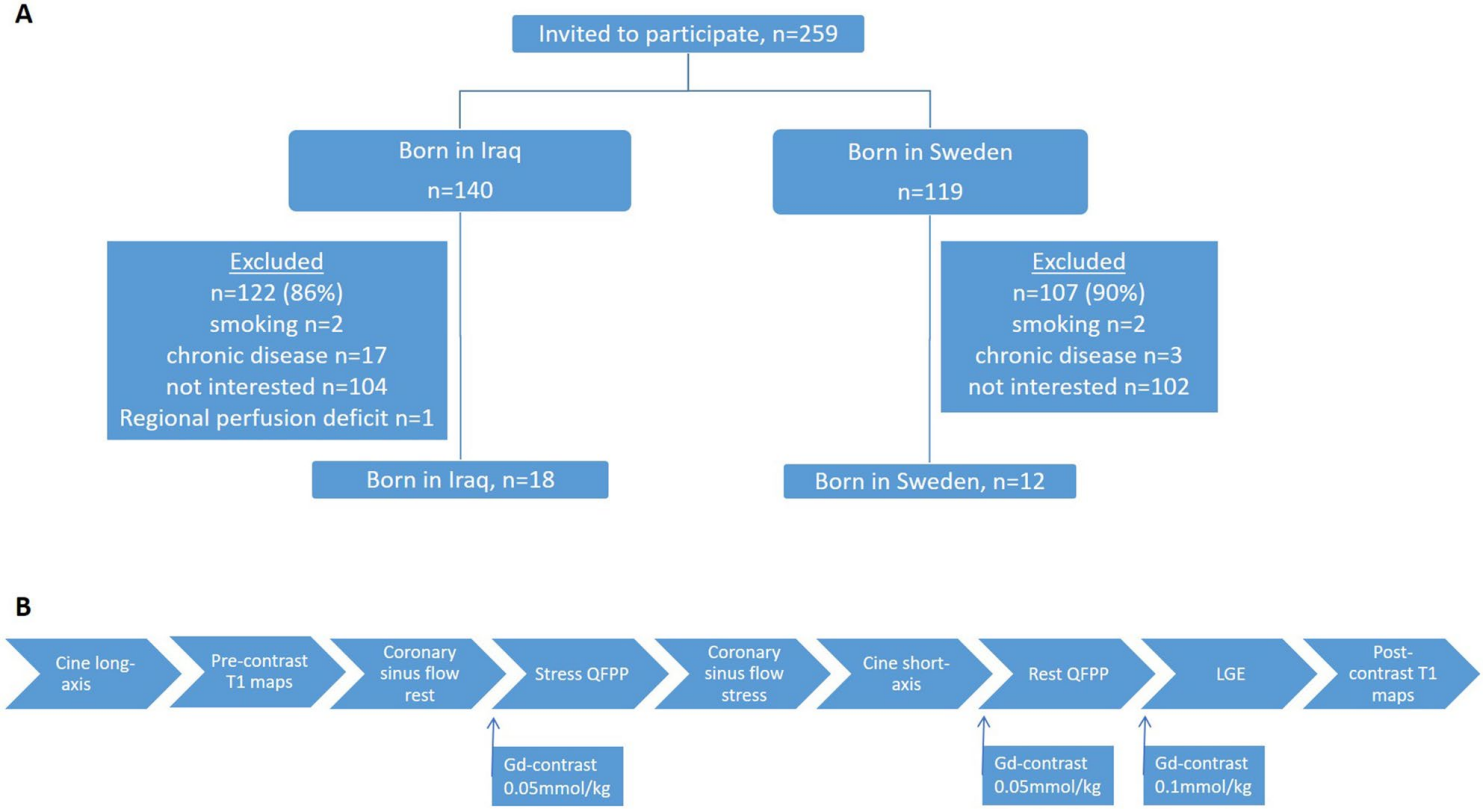
(**A**) Schematic flowchart for eligible participants, all males, performing a cardiovascular magnetic resonance study. (**B**) CMR scan protocol timeline with timings for gadolinium contrast injections.

The study was approved by the Regional Ethics Committee at Lund University Sweden (Dnr 2015/507) and conformed to the Declaration of Helsinki. Written informed consent was given by all participants. A subset of male participants in the previous MEDIM cohort without any signs or established risk factors for CVD were invited to participate in the study. The cohort has been described in detail previously^14^. Briefly, the MEDIM cohort is a cross-sectional study conducted 2010–2012 including 2155 Iraqi or Swedish born residents of Malmö 30–75 years of age. In total 259 healthy, never smokers, non-obese males without CVD risk factors were identified from the baseline study and invited by mail to participate in the present study. A total of 18 Iraqi born and twelve native Swedish born males, of Caucasian origin and none had parents originating outside of Europe, fulfilled inclusion criteria and accepted to participate in this CMR sub-study, see Fig. 1A. Exclusion criteria included known CVD, diabetes, obesity, kidney disease, history of asthma, smoking or any active medication. Also, subjects with clear regional perfusion deficits on CMR were excluded. Subjects were recruited between 2017 and 2019 and examined at Lund University Hospital, Lund, Sweden. All patients underwent rest and adenosine stress CMR and adhered to a 24-h caffeine restriction prior to scanning.

### Baseline characteristics and blood samples

Prior to the CMR exam, height, weight, waist circumference and blood pressure (in the supine position) were assessed. Fasting glucose, hemoglobin A1c, creatinine, cholesterol, triglyceride, high-density lipoprotein, low-density lipoprotein and apolipoprotein B/A1 in serum and urine albumin/creatinine were analyzed fasting as previously described^5,14^. Homeostasis model assessment (HOMA) was used to estimate both insulin resistance (HOMA-IR) and beta cell function (HOMA-β)^5^. An electrocardiogram was acquired prior to the CMR exam.

For objectively calculating the ten year risk of fatal CVD of study participants, the risk scoring systems “Framingham risk score for hard coronary heart disease”^15^ and the “systematic coronary risk evaluation (SCORE)”^16^ were used including the variables sex, age, systolic blood pressure, total cholesterol and smoking status.

### CMR image acquisition

All images were acquired on a Magnetom Aera 1.5 T CMR system (Siemens Healthcare, Erlangen, Germany), see Fig. 1B for an overview of the CMR protocol.

#### Left ventricular volumes and function

Left ventricular volumes and function were assessed by cine imaging using a steady-state free precession sequence (SSFP) in breath hold both in short-axis and long-axis projections (2-, 3- and 4-chamber views). Typical imaging parameters included repetition time (TR) = 2.7 ms, echo time (TE) = 1.2 ms, flip angle 60°, spatial resolution 1.5 × 1.5 × 8 mm with no slice gap and field of view (FOV) 270 × 320 mm^2^.

#### Quantitative first pass perfusion

A basal, a mid-ventricular and an apical short-axis image were acquired at rest and during adenosine stress (Adenosin, Life Medical AB, Stockholm, Sweden, 140 µg/kg/min infusion) using qFPP imaging during administration of an intravenous bolus of contrast agent (0.05 mmol/kg, infusion rate 4 ml/s, Gadobutrol, Gadovist, Bayer AB, Solna, Sweden). Stress images were acquired first during 60 heart beats, starting three minutes after initiation of intravenous adenosine infusion and rest images were acquired approximately 15 min later. Measurements in low-resolution proton density images optimized for high gadolinium concentration in the LV blood pool were used to calculate the arterial input function^10^. After motion correction and conversion of signal intensities to gadolinium concentration, myocardial perfusion in ml/min/g was derived on a per-pixel basis^10,13^. Typical imaging parameters were: SSFP single shot readout, TE 1.0 ms, TR 2.5 ms, flip angle 50°, FOV 360 × 270 mm^2^, slice thickness 8.0 mm, parallel acquisition technique factor 3, acquisition time per single shot slice 142 ms and saturation delay 105 ms.

#### Coronary sinus flow

Images of the coronary sinus were acquired at rest and during adenosine stress for quantification of global perfusion. Rest images were acquired first and stress images were acquired immediately after acquisition of the qFPP images, approximately 5 min later. A breath-hold phase-contrast CMR with retrospective ECG triggering was used. Typical imaging parameters were: TR 5 ms, TE 2.8 ms, flip angle 20°, parallel imaging factor 2, a reconstructed spatial resolution of 1.6 × 1.6 × 8 mm and velocity encoded (VENC) factor 80 cm/s for rest and 120 cm/s for stress.

#### Fibrosis and extracellular volume

A modified Look-Locker (MOLLI) T1 sequence was used to generate T1 maps before and after gadolinium contrast agent administration. An inline extracellular volume map (ECV-map) was created after manual input of the hematocrit. Three short axis-slices were acquired at a basal, mid-ventricular and apical level. Macroscopic fibrosis was studied using a free breathing, motion corrected, late gadolinium enhancement (LGE) sequence acquired both in short and long axis projections. The inversion time was chosen to null remote myocardium. Specific parameters for the LGE sequence were: TR 2.8 ms, TE 1.2 ms, flip angle 50°, FOV 360 × 270 mm, resolution 1.4 × 1.4 × 8 mm, no slice gap.

### CMR image analysis

All images were analyzed using the software Segment (v2.0 R5378), Medviso AB, Lund, Sweden)^17^, with the observer blinded to subject identity. For all images, endo- and epicardial borders were manually delineated. Left ventricular (LV) volumes, ejection fraction and left ventricular mass (LVM) were quantified from the short-axis cine stack. Interobserver variability for LVM was analyzed in six Iraqi and six Swedish controls. Rate pressure product (RPP) was calculated as heart rate × systolic blood pressure for both rest and stress.

#### Quantitative first pass perfusion and ECV

For qFPP and ECV images region of interest (ROI) were drawn 10% away from the endo- and epicardial borders to avoid inclusion of blood pool or extra-cardiac structures. Myocardial perfusion (ml/min/g)^10,13^ and ECV^11,12^ (%) were assessed manually by drawing a ROI in each short-axis slice. The absolute perfusion could then be extracted directly from the ROI as each pixel contained information on absolute perfusion. Myocardial perfusion and ECV were assessed globally by averaging all acquired short-axis slices. Each short-axis slice in qFPP images at rest and stress was also divided as endocardial (inner 50%) and epicardial (outer 50%) regions. Resting perfusion was corrected for rate pressure product (RPP) as resting perfusion × 10,000/((heart rate at rest) × (systolic blood pressure at rest). Myocardial perfusion reserve (MPR) was calculated as the ratio of stress to rest perfusion and MPR using RPP corrected resting perfusion was also calculated. Transmural endocardial-to-epicardial MPR gradients were also calculated. Recently, a cutoff of global stress qFPP < 2.25 ml/g/min was proposed to be suggestive of microvascular disease^19^. We also calculated the myocardial perfusion per mass of myocytes as qFPP/(1-ECV).

#### Sinus coronary flow

Global myocardial perfusion (ml/min/g) was also quantified as CSF (ml/min)/LVM (g)^20^. LVM for CSF quantification was calculated by including papillary muscles and trabeculae by visual thresholding. Interobserver variability for CSF at rest and stress was analyzed in six Iraqi and six Swedish controls and for the addition of papillary muscles and trabeculae to LVM in all subjects.

#### Late gadolinium enhancement

Focal fibrosis was assessed on short-axis LGE images by the EWA (expectation maximization weighted intensity a priori information) algorithm [21].

### Statistical analysis

Continuous data are expressed as mean ± standard deviation (SD). Mean values between groups were assessed using Fischer’s exact test, paired or unpaired t-test as appropriate in normally distributed data. The relationship between continuous variables were assessed with Pearson’s correlation coefficient. Bias according to Bland–Altman was used to compare qFPP and sinus coronary flow and for interobserver analysis. Univariable association with myocardial perfusion for covariables that differed between groups was analyzed using linear regression. All statistical analyses were performed using IBM SPSS Statistics (IBM SPSS Statistics 23, IBM, New York, USA) and Graph Pad Prism 7.0 software (Graph Pad Software, Inc., La Jolla, CA, USA). Differences with a *P* value < 0.05 were considered statistically significant.

## Results

### Study population

**Figure 2:**
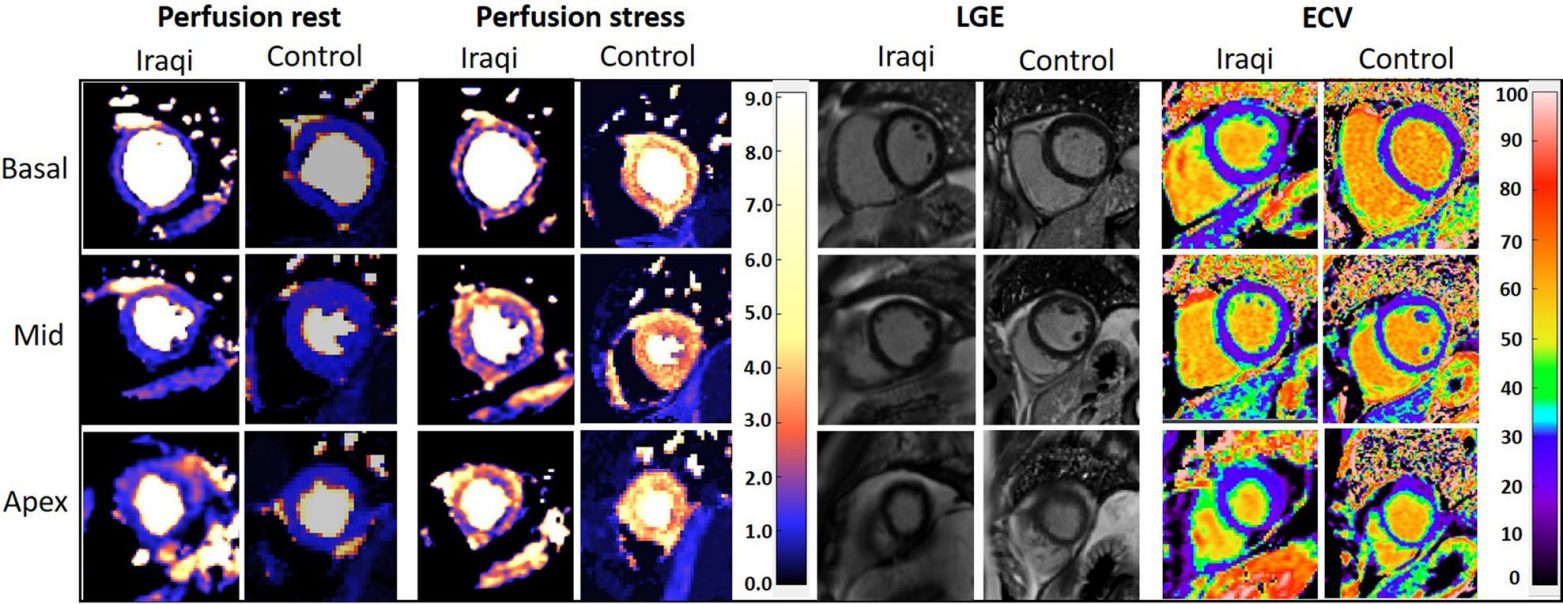
Example of quantitative first pass perfusion at rest and during adenosine stress, late gadolinium enhancement (LGE) and extracellular volume (ECV) CMR images from one Iraqi and one Swedish participant. Note the perfusion defect in the Iraqi subject at stress. No corresponding LGE or increased ECV is seen.

In one Iraqi and one Swedish control subject, resting perfusion images were not acquired, however stress images were included in the analysis. In two Iraqi subjects and one Swedish control only two slices qFPP images and T1 maps were acquired. One Iraqi subject with a regional inferior perfusion deficit was excluded. Table 1 shows baseline characteristics for all subjects. High-density lipoprotein and apolipoprotein A1/B were lower in the Iraqi group (P < 0.05) whereas HOMA-β was higher (P < 0.05) compared to the Swedish group, the latter indicating a higher level of insulin secretion. Figure 2 shows representative myocardial perfusion images at rest and stress, corresponding LGE images and extracellular volume maps. Hemodynamics and CMR imaging volumetrics for all subjects are shown in Table 2. Mean RPP were similar for Iraqi males and Swedish healthy controls at rest (7026 ± 1489 vs 7291 ± 2573, *P* = 0.72) and stress (10403 ± 2563 vs 11016 ± 1437, *P* = 0.46). The difference between stress and rest RPP did not differ between groups (3377 ± 1609 vs 3724 ± 3077, *P* = 0.68) indicating similar hemodynamic responses to adenosine stress.

**Table 1:** Baseline characteristics of study participants.

	Iraqi born males	Swedish born males	*P*
**General**			
N (males%)	18 (100)	12 (100)	> 0.99
Age (years)	47 ± 10	52 ± 12	0.18
Body mass index (kg/m^2^)	26 ± 2	25 ± 2	0.65
Waist circumference (cm)	95 ± 7	90 ± 6	0.77
**Laboratory tests**			
Fasting glucose (mmol/L)	5.6 ± 0.5	5.8 ± 0.6	0.84
HbA1c (mmol/mol)	35 ± 4	34 ± 3	0.91
Creatinine (umol/L)	81 ± 9	86 ± 14	0.24
Urine-albumine/creatinine (mg/mmol)	0.2 ± 0.3	0.3 ± 0.2	0.89
Total cholesterol (mmol/L)	4.9 ± 1.3	4.7 ± 0.6	0.75
Triglycerides (mmol/L)	1.6 ± 0.7	1.0 ± 0.6	0.06
High-density lipoprotein (mmol/L)	1.1 ± 0.3	1.5 ± 0.3	**0.02**
Low-density lipoprotein (mmol/L)	3.8 ± 0.8	3.2 ± 0.6	0.08
APO-B/APO-A1	1.0 ± 0.3	0.7 ± 0.1	**0.01**
HOMA-IR	2.1 ± 0.9 (n = 13)	1.7 ± 0.6	0.21
HOMA-β	83 ± 45 (n = 13)	55 ± 13	**0.004**
**Electrocardiogram**			
Sinus rhythm (%)	18 (100%)	12 (100%)	> 0.99
Normal electrocardiogram (%)	18 (100%)	12 (100%)	> 0.99

*HbA1c* hemoglobin A1c, *APO-B* apolipoprotein-B, *APO-A1* apolipoprotein-A1, *HOMA-IR* homeostatic model assessment for insulin resistance, *HOMA-β* homeostatic model assessment of β-cell function.

Significant values are given in bold.

**Table 2:** Hemodynamic and cardiovascular magnetic resonance imaging characteristics.

	Iraqi born males	Swedish born males	*P*
HR rest (bpm)	61 ± 12	65 ± 9	0.61
HR stress (bpm)	93 ± 22	93 ± 15	0.83
Systolic BP rest (mmHg)	113 ± 8	125 ± 17	0.35
Diastolic BP rest (mmHg)	65 ± 5	68 ± 10	0.66
Systolic BP stress (mmHg)	111 ± 7	120 ± 15	0.42
Diastolic BP stress (mmHg)	63 ± 7	64 ± 9	0.53
EF (%)	59 ± 5	63 ± 6	0.10
LVM (g)	99 ± 14	116 ± 7	**0.006**
LVMI (g/m^2^)	51 ± 7	56 ± 8	0.10
LVSV (ml)	95 ± 16	108 ± 21	0.09
LVSVI (ml/m^2^)	50 ± 16	52 ± 10	0.39
LVEDV (ml)	162 ± 22	173 ± 37	0.40
LVEDVI (ml/m^2^)	85 ± 11	84 ± 17	0.75
LVESV	66 ± 12	67 ± 19	0.44
LVESVI (ml/m^2^)	35 ± 7	33 ± 9	0.72

*HR* heart rate, *BP* blood pressure, *EF* ejection fraction, *LVM* left ventricular mass, *LVMI* left ventricular mass indexed, *LVSV* left ventricular stroke volume, *LVSVI* left ventricular stroke volume indexed, *LVEDV* left ventricular end-diastolic volume, *LVEDVI* left ventricular end-diastolic volume indexed, *LVESV* left ventricular end-diastolic volume, *LVESVI* left ventricular end-diastolic volume indexed.

Significant values are given in bold.

Framingham risk score for coronary heart disease was similar for Iraqi and Swedish males (4.0% vs 4.3%, *P* = 0.75) reflecting low CVD risk within the next decade in both Iraqi and Swedish born men. The SCORE risk score showed similar results (1.3% vs 1.5%, *P* = 0.79).

### Myocardial perfusion

#### Quantitative first pass perfusion

**Figure 3:**
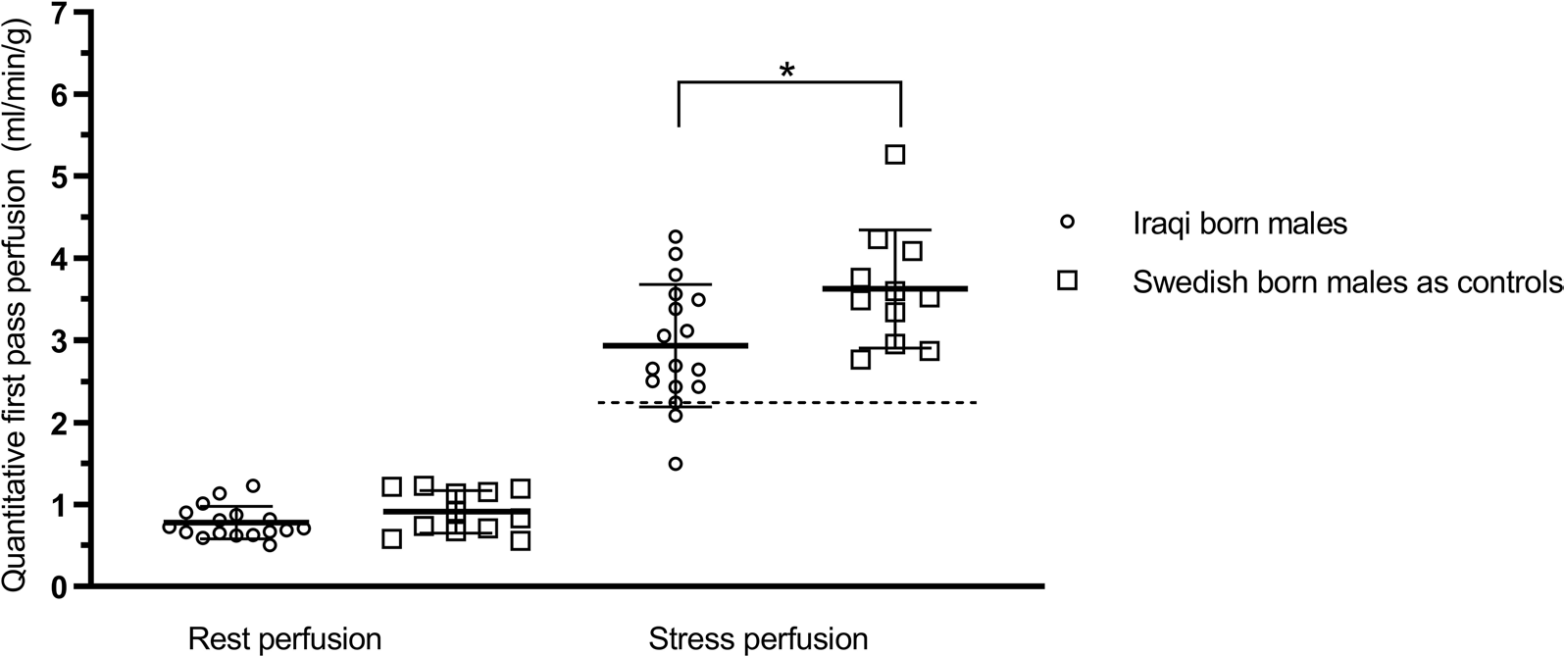
Global quantitative first pass perfusion in Iraqi versus Swedish born males at rest and during adenosine. Perfusion during adenosine was significantly lower in Iraqi males but there was no difference in myocardial perfusion at rest. If a cutoff 2.25 ml/min/g for a decreased absolute perfusion is used (dashed horizontal line) three Iraqi subjects demonstrate signs of microvascular disease. Error bars denote mean ± SD.

Global flow from qFPP analysis demonstrated no statistically significant difference between groups in resting perfusion (0.8 ± 0.2 vs 1.0 ± 0.4 ml/min/g, *P* = 0.38) or RPP corrected resting perfusion (1.2 ± 0.5 vs 1.1 ± 0.2 ml/min/g × [mmHg × bpm/10^4^]^−1^, *P* = 0.59). However, perfusion during adenosine was lower in Iraqi males (2.9 ± 0.7 vs 3.5 ± 0.7 ml/min/g. *P* = 0.02), see Fig. 3. If a cutoff of 2.25 ml/min/g for a decreased absolute perfusion was used (dashed horizontal line in Fig. 3) three Iraqi subjects demonstrated signs of microvascular disease. MPR did not show any statistically significant difference between groups (3.9 ± 1.0 vs 4.3 ± 1.2 ml/min/g, *P* = 0.42) or with RPP corrected resting perfusion (2.8 ± 1.0 vs 3.3 ± 0.8 ml/min/g × [mmHg × bpm/10^4^]^−1^, *P* = 0.14). There was also a significant difference between Iraqi and Swedish subjects in qFPP per mass of myocytes (4.0 ± 1.0 vs 4.8 ± 1.0 ml/min/g, *P* = 0.03).

#### Transmural perfusion gradient

In the Iraqi group, using qFPP, the endocardium-to-epicardium ratio at stress was lower compared to rest (0.91 ± 0.09 vs 1.03 ± 0.08 ml/min/g, *P* = 0.002). In the Swedish control group there was also a lower transmural perfusion gradient during stress (0.97 ± 0.04 vs 1.06 ± 0.10 ml/min/g, *P* = 0.001). There was no statistical significant difference in endocardial-to-epicardial qFPP stress ratio between the Iraqi and Swedish control group (P = 0.07), nor endocardial-to-epicardial MPR ratio (0.89 ± 0.09 vs 0.96 ± 0.07 *P* = 0.17).

#### Sinus coronary flow

**Figure 4:**
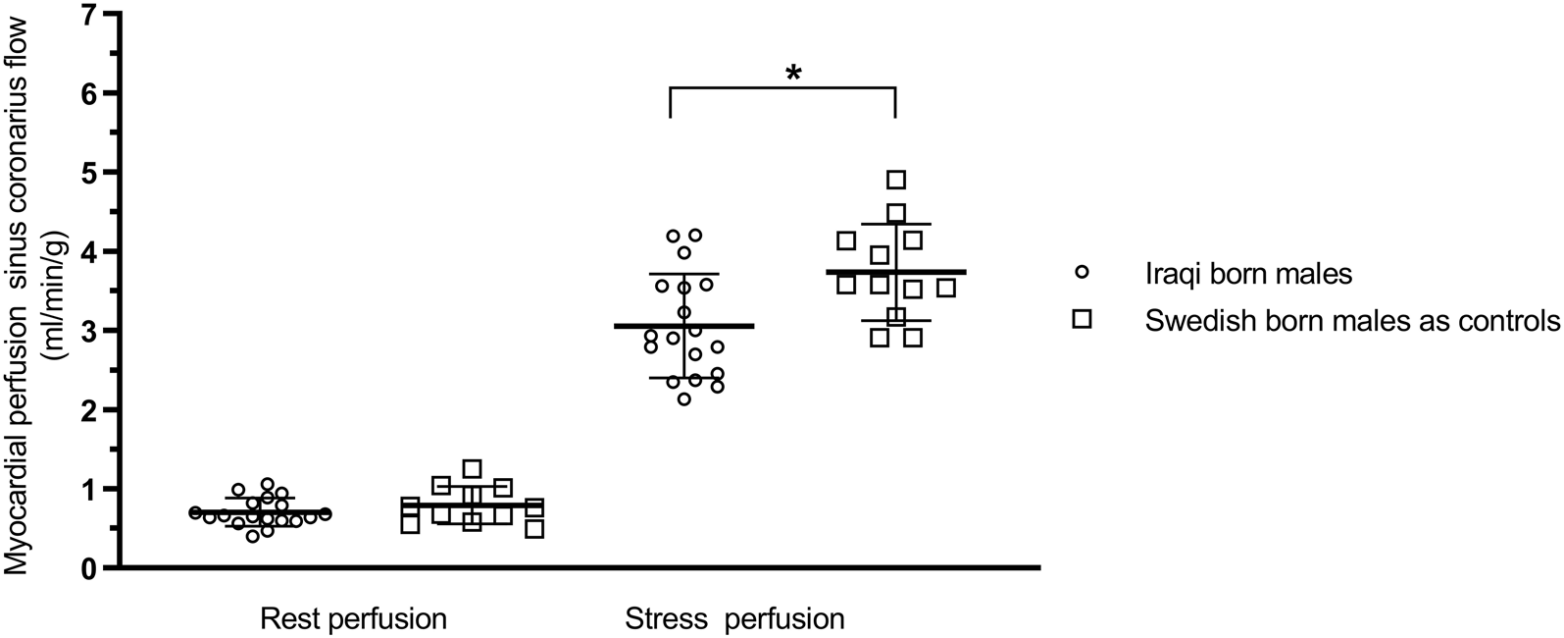
Global myocardial perfusion using sinus coronary flow analysis for Iraqi versus Swedish born males at rest and during adenosine. Myocardial perfusion during adenosine was significantly lower in Iraqi born men. Error bars denote mean ± SD.

**Figure 5:**
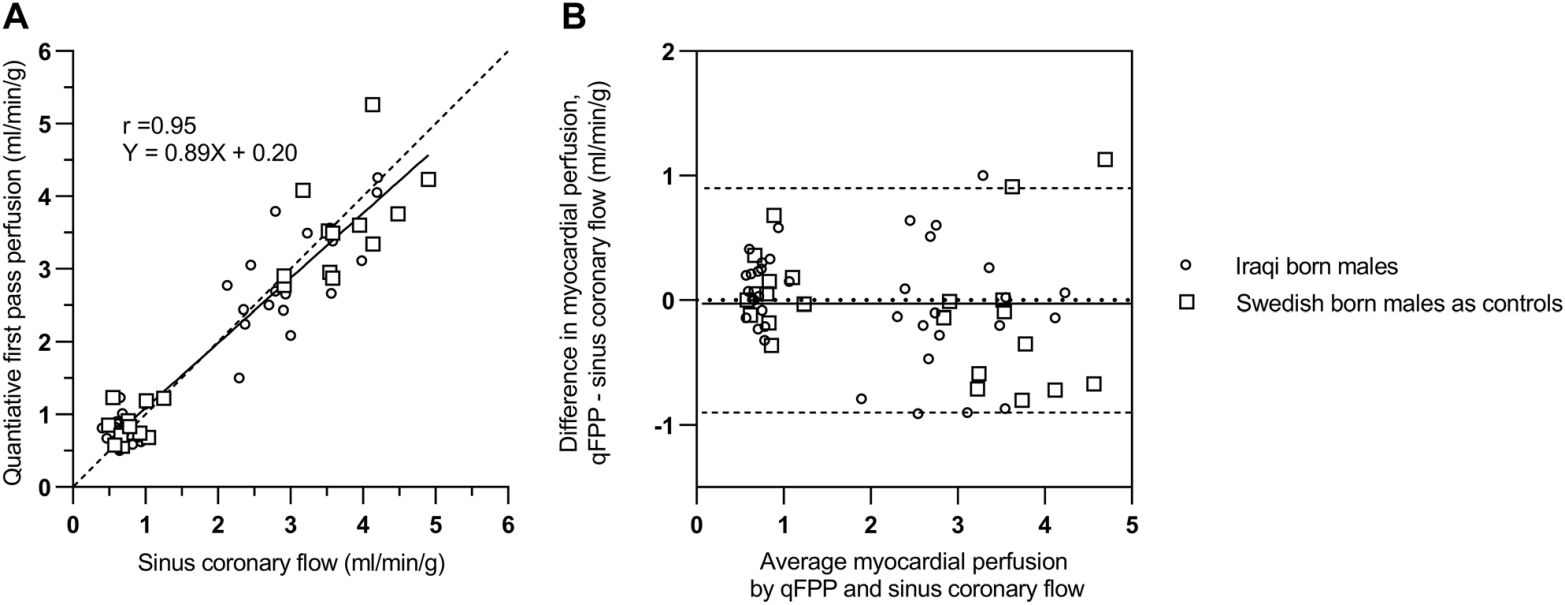
The relationship between global quantitative first pass perfusion (qFPP) and coronary sinus flow at rest and stress in Iraqi males (open circles) and Swedish born males as controls (open squares). (**A**) Global qFPP plotted against coronary sinus flow with the dashed lined representing the line of identity and solid line representing linear regression (r = 0.95, y = 0.89x + 0.20). (**B**) Agreement between qFPP and sinus coronary flow, bias 0.02 ± 0.5 ml/min/g. Dashed lines represent ± 2SD and solid line represent bias.

Global myocardial perfusion assessed by sinus coronary flow demonstrated similar results as qFPP with no difference at rest between groups (0.7 ± 0.2 vs 0.8 ± 0.2 ml/min/g. *P* = 0.21) or using RPP corrected resting perfusion (1.0 ± 0.3 vs 1.0 ± 0.2 ml/min/g. *P* = 0.82) but a lower perfusion during adenosine in the Iraqi group (3.0 ± 0.2 vs 3.7 ± 0.6 ml/min/g. *P* = 0.01), see Fig. 4. MPR did not show any statistically significant difference between groups (4.6 ± 1.3 vs 4.8 ± 1.2 ml/min/g, *P* = 0.63) or with RPP corrected resting perfusion (3.2 ± 1.2 vs 3.8 ± 1.0 ml/min/g × [mmHg × bpm/10^4^]^−1^, *P* = 0.14). The relationship between global qFPP and coronary sinus flow at rest and stress demonstrated a correlation (r) of 0.95 and bias of 0.02 ± 0.5 ml/min/g, see Fig. 5.

Interobserver variability of coronary sinus flow at both rest and stress and LVM in a subset of study participants (n = 12) was 0.1 ± 0.2 ml/min/g and 1 ± 2 g respectively and for inclusion of papillary muscles and trabeculae to LVM − 4 ± 4 g.

None of the variables insulin secretion (HOMA-β), HDL or Apo B/A1 were associated with stress perfusion using qFPP (β_HOMA-β_ = 0.094, β_HDL_ = 0.36, β_APOB/A1_ = 0.29, *P* = 0.1–0.5) or sinus coronary flow (β_HOMA-β_ = 0.091, β_HDL_ = 0.39, β_APOB/A1_ = 0.32, *P* = 0.1–0.5).

### Fibrosis and extracellular volume

LGE was not present in any subject. Extracellular volume did not differ between the Iraqi and Swedish group (27 ± 3 vs 26 ± 2%, *P* = 0.76).

## Discussion

This study demonstrates that in healthy males, myocardial perfusion by quantitative first pass perfusion and by coronary sinus flow was lower in Iraqi compared to Swedish born controls, suggesting impaired microvascular function. Despite the absence of clinical cardiovascular risk factors, early signs of CVD are present in Middle Eastern immigrants.

Two independent methods, qFPP and sinus coronary flow, were used for assessing myocardial perfusion and showed similar results and good agreement. All subjects had normal LV ejection fraction and volumes. None of the subjects demonstrated any focal fibrosis on LGE imaging and ECV values were similar between groups and within in normal range^22^. Thus, myocardial perfusion provided pathophysiological information not evident on either functional, LGE or ECV imaging. Further, these data suggest that the CVD scoring systems may be insufficient in detecting Middle Eastern immigrants at risk for CVD.

Our results in our control group on rest and stress qFPP are in line with previously published data from Nickander et al. (rest 0.80 ± 0.17 and stress 3.2 ± 0.64 ml/min/g)^23^ and Kellman et al*.* (0.95 ± 0.16 and 3.4 ± 0.39 ml/min/g)^10^. The qFPP used in the current study has shown good agreement compared to PET^13^. The results for sinus coronary flow estimates of myocardial perfusion in our control group were also similar to earlier studies by Carlsson et al.^24^. Coronary sinus flow can assess global left ventricular perfusion without the use of contrast medium administration, which can be beneficial, and can be used to test the efficacy of drug treatment^25^, to predict outcome in patients with heart failure with preserved ejection fraction^26^, with coronary artery disease^27^ and diabetes respectively^28^.

Using a cutoff for global qFPP of 2.25 ml/min/g three Iraqi subjects showed signs of microvascular dysfunction. It has been proposed that a transmural myocardial MPR ratio < 0.72^29^ and < 0.8^30^ from endocardium-to-epicardium can be suggestive of microvascular dysfunction in microvascular angina patients using semi-quantitative myocardial perfusion. With these cut-offs three Iraqi subjects showed signs of microvascular dysfunction (all with ratios < 0.72). However, in our study, patients were asymptomatic and a different CMR perfusion technique was used making a comparison somewhat difficult. In addition, it has been debated if a single cut-off value for transmural gradients should be used given that normal physiological mechanisms influence the endocardium-to epicardium MPR gradient^31^.

Previously, first-generation Iraqi immigrants living in Sweden have shown to be more insulin resistant^5^, have a higher prevalence of type 2 diabetes^14^ but paradoxically an overall beneficial lipid profile than native Swedes^32^. The Iraqi group in our study had higher beta-cell function assessed by HOMA-β, suggesting early compensatory insulin secretion for the higher insulin resistance. Also, the Iraqi group had a more unfavorable lipid profile with lower levels of HDL and higher APO B/A1. However, differences in stress perfusion were not associated with either HOMA-B or lipid profile. We could not find any cardiovascular risk factors explaining the variation in myocardial perfusion across males of Middle Eastern and Caucasian origin. From the same MEDIM cohort as the current study is based on two studies on differences in fat metabolism have recently demonstrated that (1) healthy Iraqi male immigrants displayed a higher triglyceride peak and a delayed postprandial triglyceride clearance compared with Swedes after an oral fat tolerance test^33^ and (2) Iraqi-born men presented a more favorable abdominal fatty-acid composition compared to Swedish-born men using MRI of the abdomen^34^.

The results in this study should also be interpreted in the light of both groups being a priori cardiovascular healthy, without any symptoms of CVD and with normal ten-year risk scores for myocardial infarction or cardiovascular death. All subjects had normal 12-lead electrocardiograms. Thus, according to standard assessment for CVD risk none of the subjects in the Iraqi group would be expected to have signs of myocardial microvascular disease. From a clinical perspective, assessment of cardiometabolic risk factors is crucial in estimating CVD risk. Previous CVD risk calculators are primarily based on Caucasian populations, but our data indicate these scores may be insufficient when estimating CVD risk in other ethnic populations.

The results of this study should be interpreted in the light of some limitations. There was a limited number of subjects in this study and the results should be confirmed in larger cohorts. None of the subjects performed an assessment of the coronary epicardial vessels. No females were included in this study and therefore results cannot be generalized to both sexes. Also recently, it was shown by Nickander et al*.* that there is a difference in myocardial perfusion between sexes^23^ which warrant further caution in interpretation of results. A high number of invited participants declined to participate even though they got follow up phone calls and contact with the primary investigator. The high dropout is a potential inclusion bias to the study.

This study on healthy Iraqi immigrants living in Sweden with no established risk factors for coronary artery disease or microvascular disease demonstrate significantly lower myocardial perfusion during adenosine stress compared to Swedish controls. Our results demonstrate the value of non-invasive CMR imaging to detect potential early alterations to the myocardial microvascular system. Despite the absence of clinical cardiovascular risk factors, early signs of CVD may be present in Middle Eastern immigrants. Thus, myocardial perfusion provided incremental pathophysiological information to both functional, LGE and ECV imaging. Also, CVD scoring systems may be insufficient in detecting people of Middle Eastern origin at risk for CVD. This calls for future altered preventive strategies in detecting cardiovascular disease in this high-risk population.

## Data Availability

The datasets used and/or analysed during the current study are available from the corresponding author on reasonable request.
